# Temporary tracheostomy required as an infant may be a risk factor for future centrally mediated disordered sleep ventilation

**DOI:** 10.1186/1743-8454-7-S1-S51

**Published:** 2010-12-15

**Authors:** Robert Adderley, David Wensley

**Affiliations:** 1 Critical Care Services The University of British Columbia Room 2L5 British Columbia's Children's Hospital 4480 Oak Street Vancouver, British Columbia V6H 3V4, Canada

## Background

Children with spina bifida experience lifelong complex medical issues. The problems of locomotion, and neurogenic bowel and bladder are well appreciated, and the necessity of shunting obstructive hydrocephalus is equally well known. There are however, serious, less common problems associated with control of ventilation. Most common is vocal cord dysfunction, with unilateral or bilateral vocal cord paresis, and as a consequence, upper airway obstruction. Rarer still, are patients with life threatening breath holding spells, central apnea, or mixed central and obstructive apnea. In many cases, surgical decompression of the posterior fossa can result in return of vocal cord function, and relief of obstructive apnea. In a few cases tracheostomy is required. With a Chairi II malformation, the upper medulla, where the nuclei of cranial nerves IX and X lie close to the rostral tracts of the respiratory centre, may have an abnormal and tenuous blood supply. The medulla may be compromised by bony pressure or by compromise of the blood supply (herniation or chronic arachnoiditis).

## Materials and methods

The Spinal Cord Program at British Columbia’s Children’s Hospital has cared for 956 patients since 1982. Over the same period, the Home Tracheostomy Care/ Home Ventilation Program has cared for 346 children, eight with spinal dysrhaphism. Two girls with meningomyelocoele and Arnold Chiari type II malformations, who had required tracheostomies as infants, presented as adolescents with symptoms suggestive of disordered sleep ventilation. Both had required a tracheostomy despite timely posterior fossa decompression, but over time (years), gag and vocal cord function returned, and they were successfully decannulated. Patient 1, with a lumbosacral meningomyelocoele was referred to the Home Ventilation Program at fourteen years of age when her mother, a registered nurse reported erratic breathing at night, with weight loss, and deteriorating school performance. Patient 2, with a lumber meningomyelocoele presented at fifteen years of age complaining of daytime somnolence, but denied morning headaches. In both cases polysomnography showed a similar, chaotic pattern of respiration with frequent arousals and severely fragmented sleep. Computerized Tomography showed no change in ventricular size in either case.

## Results

Both patients were started on nighttime noninvasive ventilation (NIPPV) with rapid resolution of symptoms.

## Conclusions

Children with meningomyelocoele, particularly those who appear to have had resolution of infantile bulbar dysfunction, may present later in life with severely disordered sleep ventilation, and warrant careful lifelong followup, with a low threshold for polysomnography and institution of ventilatory support.

